# Cardiovascular Therapeutics at the Crossroads: Pharmacological, Genetic, and Digital Frontiers

**DOI:** 10.3390/ph18111703

**Published:** 2025-11-10

**Authors:** Erica Vetrano, Alfredo Caturano, Davide Nilo, Giovanni Di Lorenzo, Giuseppina Tagliaferri, Alessia Piacevole, Mariarosaria Donnarumma, Ilaria Iadicicco, Sabrina Picco, Simona Maria Moretto, Maria Rocco, Raffaele Galiero, Vincenzo Russo, Raffaele Marfella, Luca Rinaldi, Leonilde Bonfrate, Ferdinando Carlo Sasso

**Affiliations:** 1 Department of Advanced Medical and Surgical Sciences, University of Campania Luigi Vanvitelli, 80138 Naples, Italy; erica.vetrano@unicampania.it (E.V.); nilodavide@gmail.com (D.N.); giuann86@gmail.com (G.D.L.); giuseppina.tagliaferri@gmail.com (G.T.); alessia.piacevole@studenti.unicampania.it (A.P.); mariarosaria.donnarumma@unicampania.it (M.D.); ilariaiad@gmail.com (I.I.); sabrina.picco1@studenti.unicampania.it (S.P.); simonamaria.moretto@studenti.unicampania.it (S.M.M.); maria.rocco@studenti.unicampania.it (M.R.); raffaele.galiero@unicampania.it (R.G.); raffaele.marfella@unicampania.it (R.M.); ferdinandocarlo.sasso@unicampania.it (F.C.S.); 2 Department of Human Sciences and Promotion of the Quality of Life, San Raffaele Roma University, 00166 Rome, Italy; leonilde.bonfrate@uniroma5.it; 3 Sbarro Institute for Cancer Research and Molecular Medicine, Center for Biotechnology, College of Science and Technology, Temple University, Philadelphia, PA 19122, USA; v.p.russo@libero.it; 4 Division of Cardiology, Department of Medical Translational Sciences, University of Campania Luigi Vanvitelli, 80138 Naples, Italy; 5 Department of Medicine and Health Sciences “Vincenzo Tiberio”, Università degli Studi del Molise, 86100 Campobasso, Italy; luca.rinaldi@unimol.it

**Keywords:** cardiovascular therapeutics, PCSK9 inhibitors, inclisiran, SGLT2 inhibitors, GLP-1 receptor agonists, tirzepatide, gene therapy, nanomedicine

## Abstract

Therapeutic innovation in cardiovascular medicine is rapidly overcoming the limitations of conventional strategies, providing more targeted, durable, and multidimensional solutions. Key advances include next-generation lipid-lowering agents such as PCSK9 inhibitors, inclisiran, and bempedoic acid, as well as metabolic drugs like SGLT2 inhibitors, GLP-1 receptor agonists, and dual GIP/GLP-1 agonists, which offer cardiovascular and renal benefits beyond glucose control. At the same time, gene therapies, RNA-based interventions, genome editing tools, and nanocarriers are paving the way for precision medicine tailored to individual patient profiles. In parallel, digital innovations, including artificial intelligence, remote monitoring, and telehealth platforms, are transforming care delivery by enhancing adherence, enabling earlier intervention, and refining risk stratification. Collectively, these developments signify a paradigm shift toward a more personalized, proactive, and systems-based model of cardiovascular care.

## 1. Introduction

Cardiovascular diseases (CVDs) remain the leading cause of mortality in Western countries, significantly impairing quality of life and imposing a substantial economic burden on healthcare systems [1]. Despite progress in diagnostic techniques and therapeutic interventions, the prevalence and impact of CVDs remain high, particularly in patients with comorbidities such as diabetes, obesity, and chronic kidney disease (CKD) [2]. This enduring burden highlights the urgent need to reassess current treatment paradigms and adopt innovative strategies that address the limitations of conventional care. For decades, the pharmacological management of CVDs has relied on cornerstone therapies, including angiotensin-converting enzyme (ACE) inhibitors for hypertension and heart failure (HF) [3], beta-blockers for angina, arrhythmias, and post-myocardial infarction care [4], and statins, which revolutionized atherosclerosis prevention by significantly lowering LDL cholesterol and reducing cardiovascular events [5]. However, a substantial proportion of patients continue to experience residual cardiovascular risk, even under optimal therapy [6]. This may stem from factors such as drug resistance, side effects, limited adherence, or the inadequacy of standardized, one-size-fits-all approaches in complex clinical contexts. In response, a shift toward a personalized and multidimensional therapeutic model is emerging [7]. Advanced therapies developed through genetic engineering, biotechnology, and digital innovation are reshaping cardiovascular medicine. Examples include Proprotein convertase subtilisin/kexin type 9 (PCSK9) inhibitors that dramatically reduce LDL cholesterol in high-risk individuals [8]; RNA-based therapies such as inclisiran, which silences PCSK9 production at the mRNA level [9]; and bempedoic acid, an oral alternative for patients who are intolerant to statins [10]. Additionally, cardiometabolic agents initially designed for glycemic control, such as SGLT2 inhibitors and GLP-1 receptor agonists, have shown substantial cardiovascular and renal benefits independent of glucose regulation [11,12]. Importantly, the landscape of innovation extends beyond pharmacotherapy. Gene therapies, genome editing technologies (e.g., CRISPR/Cas9), nanomedicine, and digital health tools are increasingly integrated into clinical practice [13,14]. These advancements support the evolution of predictive, preventive, and patient-centered care. Digital innovations, including remote monitoring, machine learning-based diagnostic tools, and wearable health technologies, are already improving adherence, enhancing early detection, and enabling individualized disease management [15,16]. This review aims to achieve two objectives: first, to provide a critical, up-to-date overview of emerging therapeutic innovations in cardiovascular care, with a focus on newly approved drugs and mechanisms of action; and second, to explore the clinical potential of advanced technologies, assessing their benefits, limitations, and future applications. In pursuit of these objectives, we reviewed original studies, systematic reviews, international guidelines, and major clinical trials published over the past two decades. Our analysis concentrates on prevalent chronic diseases such as hypercholesterolemia, HF, and type 2 diabetes (T2DM), and emphasizes therapies that are either already approved or in advanced stages of development. Ultimately, we aim to offer a comprehensive and forward-looking perspective on the evolving landscape of cardiovascular therapeutics.

## 2. Methods

Relevant literature was identified through a comprehensive search of PubMed/MEDLINE, Scopus, and Web of Science, focusing on publications between January 2003 and August 2025. Additional references were retrieved by screening bibliographies of key studies and international guidelines. Priority was given to randomized controlled trials, meta-analyses, large observational studies, translational research, and position statements from major cardiovascular societies (ESC, AHA/ACC, ADA, EAS). Preclinical and early-phase studies were included, illustrating novel mechanisms or therapeutic approaches of translational relevance. Studies not published in English, single case reports, or those lacking relevance to cardiovascular prevention and treatment were excluded.

## 3. Molecular Innovations in Lipid-Lowering Therapies

### 3.1. PCSK9 Inhibitors (Alirocumab & Evolocumab)

**Figure 1:**
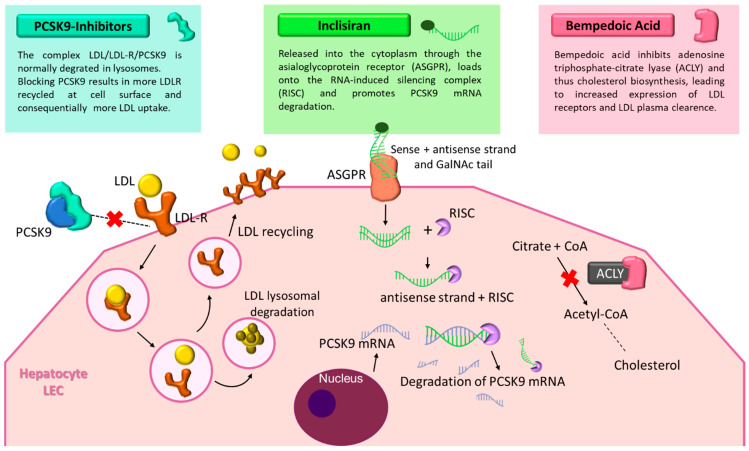
Mechanisms of novel lipid-lowering therapies: PCSK9 inhibitors, inclisiran, and bempedoic acid. The figure illustrates the distinct molecular mechanisms of three next-generation lipid-lowering agents. PCSK9 inhibitors prevent PCSK9 from binding to LDL receptors (LDL-R), allowing receptor recycling and enhanced LDL clearance. Inclisiran, a small interfering RNA conjugated with GalNAc, targets PCSK9 mRNA in hepatocytes, reducing its synthesis. Bempedoic acid inhibits ATP-citrate lyase, decreasing cholesterol biosynthesis and upregulating LDL receptor expression. Together, these agents improve lipid control and reduce cardiovascular risk. Abbreviations: PCSK9, proprotein convertase subtilisin/kexin type 9; LDL, low-density lipoprotein; LDL-R, low-density lipoprotein receptor; ASGPR, Asialoglycoprotein receptor; RISC, RNA-induced silencing complex; ACLY, adenosine triphosphate-citrate lyase; CoA, Coenzyme A; mRNA, messenger RNA; LEC, liver endothelial cell.

PCSK9 is a key regulator of cholesterol metabolism. By binding to low-density lipoprotein receptors (LDLRs) on hepatocytes, PCSK9 promotes their degradation, thereby reducing the liver’s capacity to clear LDL cholesterol from circulation (Figure 1). This mechanism has made PCSK9 an attractive therapeutic target, particularly in patients who fail to reach lipid goals with statins alone [17]. Alirocumab and evolocumab are fully human monoclonal antibodies (IgG1 and IgG2, respectively) that inhibit PCSK9 with high specificity, preventing its interaction with LDLRs and enabling receptor recycling and enhanced LDL clearance. These agents can lower LDL-C levels by up to 60–70%. Beyond lipid reduction, clinical trials have demonstrated favorable effects on vascular health. The PACMAN-AMI trial [18] showed that alirocumab, initiated early after acute myocardial infarction in combination with high-intensity statins, significantly improved plaque characteristics as assessed by intracoronary imaging at 52 weeks. Similarly, the ODYSSEY Outcomes trial [19] reported a 15% relative reduction in major adverse cardiovascular events (MACE) with alirocumab in high-risk post-ACS patients. Evolocumab has demonstrated comparable efficacy, including in patients with and without multivessel coronary artery disease [20,21]. It is administered either as doses of 140 mg biweekly or 420 mg monthly, though in specific cases such as homozygous familial hypercholesterolemia, more intensive regimens may be required [20]. These therapies are generally well tolerated, with the most frequent side effects being mild injection-site reactions. Importantly, they are not associated with muscle pain, new-onset diabetes, or cognitive impairment, concerns that limit the use of other lipid-lowering drugs. Given their efficacy and safety, PCSK9 inhibitors are increasingly incorporated into guideline recommendations and clinical practice, especially for statin-intolerant patients or those at high cardiovascular risk [22,23].

### 3.2. Inclisiran: An RNA Interference Approach

Inclisiran represents a novel therapeutic class that leverages RNA interference to lower LDL cholesterol. This small interfering RNA (siRNA) targets PCSK9 mRNA within hepatocytes, thereby reducing both intracellular and circulating PCSK9 levels [24]. The molecule is conjugated to triantennary *N*-acetylgalactosamine (GalNAc), which enhances hepatic uptake through asialoglycoprotein receptors (Figure 1). Inclisiran was approved in the European Union in December 2020 for the treatment of adult patients with primary heterozygous familial or non-familial hypercholesterolemia, or mixed dyslipidemia, as an adjunct to dietary measures. Following subcutaneous injection, inclisiran is internalized by hepatocytes, where it enters the RNA-induced silencing complex (RISC) and guides the degradation of PCSK9 mRNA. This process increases LDLR recycling and improves LDL clearance. Its long-lasting effect allows for a simplified dosing regimen: initial injections at baseline and 3 months, followed by maintenance dosing every 6 months [24]. Inclisiran has demonstrated consistent LDL-C reductions of approximately 50% across multiple phase III trials (ORION-9, -10, -11), with a safety profile comparable to that of monoclonal antibodies. In the ORION-3 extension study, it matched the LDL-C-lowering efficacy of evolocumab, with the added advantage of biannual dosing. Post hoc analyses in post-myocardial infarction populations have reinforced its potential role in secondary prevention, although outcome data on MACE are still awaited from the ongoing ORION-4 trial [4,25,26,27].

The most common adverse events are mild injection-site reactions. Inclisiran does not significantly affect liver enzymes, muscle function, or cognition, and it lacks cytochrome P450 interactions, making it particularly suitable for patients on complex medication regimens [28]. Nevertheless, its broader clinical adoption may be limited by its high cost and the current absence of definitive outcome data.

### 3.3. Bempedoic Acid

Bempedoic acid (BA) is an oral prodrug that, once activated in the liver, inhibits ATP-citrate lyase, an enzyme upstream of HMG-CoA reductase in the cholesterol biosynthesis pathway [6,29]. By reducing hepatic cholesterol production and upregulating low-density lipoprotein receptors (LDLRs), BA effectively lowers LDL-C levels (Figure 1). A distinctive feature is that its activation requires very-long-chain acyl-CoA synthetase-1 (ACSVL1), an enzyme expressed in the liver but absent in skeletal muscle. This confers a key advantage: a markedly reduced risk of myopathy or muscle-related side effects, making BA an attractive option for statin-intolerant patients [30]. In the CLEAR Outcomes trial, BA achieved a 13% reduction in MACE over 40.6 months in more than 13,000 statin-intolerant patients. Typical LDL-C reductions range from 20 to 25%, with enhanced efficacy when combined with ezetimibe, forming a potent non-statin oral regimen for patients with atherosclerotic cardiovascular disease [6,31]. Real-world evidence from a multicenter Italian study further supports its effectiveness, demonstrating substantial LDL-C reductions, high adherence (99%), and favorable tolerability in both high-risk and statin-intolerant populations [32]. Adverse events include mild hyperuricemia and anemia, while serious complications are rare [31,33]. BA does not significantly affect liver enzymes, cause muscle symptoms, or interfere with the cytochrome P450 system, making it suitable for patients with polypharmacy. Thanks to its once-daily oral dosing, muscle-sparing profile, and lower cost compared with injectable agents, BA represents a practical and accessible option for improving adherence to lipid-lowering therapy. Ongoing research is also investigating fixed-dose combinations of BA with cardiometabolic agents such as GLP-1 receptor agonists or SGLT2 inhibitors, which may further enhance its role in integrated cardiovascular prevention.

## 4. Advances in Cardiovascular and Metabolic Protection

### 4.1. SGLT2 Inhibitors: From Antidiabetic Agent to Cardio- and Nephroprotective Therapy

#### 4.1.1. SGLT2 Inhibitors in Type 2 Diabetes Mellitus

In 2008, the U.S. Food and Drug Administration (FDA) approved sodium-glucose co-transporter 2 (SGLT2) inhibitors for the treatment of hyperglycemia in patients with T2DM. Early studies confirmed their efficacy in lowering plasma glucose and improving short-term outcomes in adults with T2DM [34]. Soon after their clinical introduction, accumulating evidence revealed that their therapeutic benefits extended well beyond glycemic control, radically transforming their clinical role [35].

#### 4.1.2. Cardiovascular Protection: Heart Failure and Atherosclerotic Disease

Large cardiovascular-outcome trials, including EMPA-REG OUTCOME (empagliflozin), CANVAS and CREDENCE (canagliflozin), DECLARE-TIMI 58 and DAPA-HF (dapagliflozin), and VERTIS-CV (ertugliflozin) trials, demonstrated that SGLT2 inhibitors significantly reduce hospitalizations for HF and adverse renal events, with some also showing reductions in lowering MACE [36,37]. Meta-analyses confirm a class-wide ≈25–30% reduction in HF hospitalizations, largely independent of diabetes status [38]. These findings have established SGLT2 inhibitors as essential agents in the management of HF, regardless of ejection fraction or baseline glycemia.

#### 4.1.3. Renal Protection and Mechanisms of Benefit

Beyond cardiovascular outcomes, SGLT2 inhibitors markedly slow CKD progression, reducing renal-endpoint risk by ≈30–40% irrespective of baseline kidney function [38]. Mechanistically, they induce osmotic diuresis and natriuresis, lowering preload and afterload while decreasing interstitial and intravascular volume without significant electrolyte imbalance [39,40]. Additional renal and myocardial benefits derive from decreased intraglomerular pressure, improved renal oxygenation, the restoration of tubuloglomerular feedback, and a metabolic shift toward more energy-efficient myocardial substrates [41]. Anti-inflammatory, antifibrotic, and antioxidative actions further contribute to cardio-renal protection [40,42,43].

#### 4.1.4. Safety and Emerging Indications

SGLT2 inhibitors are generally well tolerated. The most frequent adverse effects are genital mycotic infections and mild volume depletion, while euglycemic diabetic ketoacidosis remains rare and typically preventable with appropriate patient education [38]. Importantly, these agents carry a low risk of hypoglycemia, have neutral or favorable effects on body weight and blood pressure, and can be safely combined with other cardioprotective therapies such as GLP-1 receptor agonists [41,44]. Ongoing evaluations are exploring new indications, including acute HF, HF with mildly reduced ejection fraction, and earlier CKD stages, as well as combination regimens integrating metabolic and anti-inflammatory strategies [45,46,47]. Collectively, SGLT2 inhibitors represent one of the most important therapeutic innovations of the past decade, redefining the boundaries between diabetology, nephrology, and cardiology through an integrated approach to cardio-renal-metabolic protection.

### 4.2. GLP-1 Receptor Agonists: Beyond Glucose Control Toward Cardiovascular Protection

Glucagon-like peptide-1 receptor agonists (GLP-1 RAs) have redefined the therapeutic management of T2DM, offering benefits that extend well beyond glucose lowering [48]. Acting through the incretin pathway, these agents enhance glucose-dependent insulin secretion, suppress glucagon release, delay gastric emptying, and reduce appetite, leading to both glycemic improvement and weight loss [49].

#### 4.2.1. Cardiovascular Protection

Beyond metabolic regulation, GLP-1 RAs exert anti-inflammatory, anti-atherogenic, and endothelial-protective actions that contribute to cardiovascular (CV) risk reduction [49]. Evidence from major cardiovascular outcome trials, including LEADER (liraglutide), SUSTAIN-6 and PIONEER 6 (semaglutide), REWIND (dulaglutide), and HARMONY Outcomes (albiglutide), has consistently demonstrated a 12–26% reduction in MACE in patients with T2DM at high CV risk [50]. These benefits are largely independent of glycemic control and are driven mainly by reductions in non-fatal stroke and myocardial infarction [51]. Meta-analyses confirm that GLP-1 RAs significantly lower MACE, cardiovascular mortality, and all-cause mortality compared with placebo, with comparable efficacy across subcutaneous and oral formulations [52]. Mechanistically, GLP-1 RAs improve multiple cardiometabolic pathways. They promote weight loss, lower blood pressure, improve lipid profiles, and enhance endothelial nitric oxide availability, while also attenuating oxidative stress and vascular inflammation [53]. Some experimental data suggest direct myocardial effects, including improved cardiac metabolism and protection against ischemia–reperfusion injury [54].

#### 4.2.2. Obesity and Emerging Indications

The favorable weight-reducing and metabolic effects of GLP-1 RAs have broadened their role beyond diabetes. Once-weekly formulations (semaglutide, dulaglutide, exenatide ER) and oral semaglutide have improved adherence and patient satisfaction [55,56,57]. Ongoing studies are exploring next-generation molecules with enhanced receptor affinity or dual mechanisms that extend benefits to non-diabetic populations, obesity, HF with preserved ejection fraction, and atherosclerosis prevention [58].

#### 4.2.3. Renal Protection

Recent evidence suggests that GLP-1 receptor agonists also confer direct renal benefits, complementing those of SGLT2 inhibitors. In pooled analyses of cardiovascular outcome trials, these agents slowed the decline in estimated glomerular filtration rate and reduced new-onset macroalbuminuria, independent of glycemic control or blood pressure effects [59]. The FLOW trial [60] provided the first dedicated renal-outcome evidence, showing significant reductions in kidney-disease progression and cardiovascular death in patients with T2DM and CKD. These data indicate that GLP-1 RAs may play a meaningful role in comprehensive kidney protection across the cardio-renal-metabolic continuum.

#### 4.2.4. Safety Profile

The class is generally well tolerated. Gastrointestinal adverse effects, mainly nausea and vomiting, are the most frequent, usually transient and dose-dependent. Rare events such as pancreatitis or gallbladder disease warrant vigilance, though no consistent safety signal has emerged in large trials [55]. By integrating potent metabolic control with direct vascular and cardiac benefits, they bridge the gap between endocrinology and cardiology, marking a key step toward comprehensive cardiovascular risk reduction.

### 4.3. Tirzepatide: Innovative Dual-Action Approach and Its Potential Benefits in Metabolic Control

Tirzepatide is one of the most recent pharmacological options for the treatment of obesity and T2DM [61]. Despite therapeutic advances, fewer than half of patients with T2DM achieve the target HbA1c < 7%, and approximately 50% are overweight, highlighting the need for innovative strategies in metabolic control. Unlike currently available agents, tirzepatide exerts dual agonist activity on both the glucose-dependent insulinotropic polypeptide (GIP) and glucagon-like peptide-1 (GLP-1) receptors. It is a multifunctional polypeptide based on the GIP sequence, structurally modified to also activate the GLP-1 receptor. Composed of 39 amino acids and conjugated with a fatty acid chain to prolong half-life, tirzepatide is suitable for once-weekly administration [62]. Tirzepatide displays greater affinity for the GIP receptor, hence the designation of an “unbalanced dual agonist” [63]. Notably, GLP-1 receptor activation also enhances receptor expression and reduces degradation, amplifying the therapeutic effect [63]. This dual mechanism drives systemic metabolic benefits through the receptors’ differential tissue distribution: both receptors are expressed in the pancreas and central nervous system, while GLP-1 receptors are also present in the stomach and liver, and GIP receptors in skeletal muscle and adipose tissue [64,65]. The main clinical effects are improvements in glycemic control and weight reduction, mediated by increased insulin secretion and sensitivity. These outcomes have been consistently confirmed in the SURPASS program [66,67,68,69] and the SURMOUNT program [70,71,72,73]. In the SURPASS-2 trial, tirzepatide 5 mg produced greater HbA1c reductions than semaglutide 1 mg (−2.09% vs. −1.86%) within four weeks of treatment initiation [67]. Superiority was also demonstrated over basal insulin and basal-bolus insulin regimens [68,69]. Approximately 80% of patients achieved HbA1c <7%, and about one-third achieved <6.5% [66]. In terms of weight loss, tirzepatide showed greater efficacy than semaglutide, with mean reductions of −7.8 kg versus −6.2 kg, and ≥5% weight loss achieved in more than 70% of patients [73]. Long-term cardiovascular effects are being investigated in the SURPASS-CVOT trial, which compares tirzepatide with dulaglutide in patients with T2DM at high CV risk [74,75]. Preliminary results from the SURPASS-CVOT trial have recently been reported, marking the first head-to-head cardiovascular outcomes study comparing tirzepatide (at maximum tolerated dose 5 mg, 10 mg or 15 mg) with dulaglutide (1.5 mg) in individuals with T2DM and established atherosclerotic cardiovascular disease [74]. Tirzepatide met its primary endpoint, demonstrating non-inferiority for major adverse cardiovascular events (MACE-3; HR = 0.92; 95.3% CI 0.83–1.01), while also showing significant improvements in key secondary outcomes, including reductions in all-cause mortality (HR = 0.84; 95% CI 0.75–0.94), slower eGFR decline, greater HbA1c reduction (−0.83%), and superior weight loss (−7.1%) compared with dulaglutide. Emerging data also indicate potential benefits in metabolic dysfunction associated steatotic liver disease and metabolic dysfunction-associated steatohepatitis, with reductions in hepatic steatosis and liver fat content observed in phase II studies. The most common adverse events are gastrointestinal, similar to those seen with GLP-1 RAs. Interestingly, GIP receptor activation may mitigate nausea through an antiemetic mechanism mediated by GABAergic neurons in the brainstem, potentially improving tolerability [76]. Tirzepatide has been approved for the treatment of T2DM and obesity in both the United States and the European Union. In Italy, it became available in late 2024 and has been reimbursed by the National Health System since early 2025. Given its dual mechanism and robust efficacy in both glycemic control and weight management, tirzepatide represents a major therapeutic advance in metabolic disorders. These findings suggest that tirzepatide preserves the cardioprotective benefits of GLP-1 receptor agonists while offering additional metabolic and renal advantages, reinforcing its potential as a preferred therapy for patients with T2DM and high cardiovascular risk.

**Figure 2:**
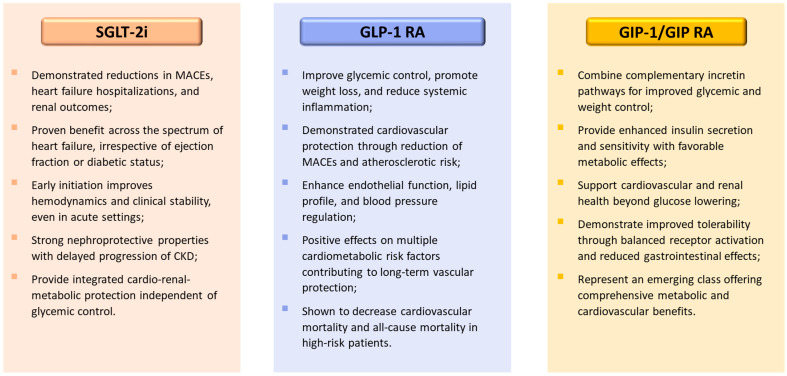
Comparative overview of cardiometabolic drug classes: SGLT-2 inhibitors, GLP-1 receptor agonists, and dual GIP/GLP-1 receptor agonists. The figure summarizes the principal mechanisms and class-wide clinical effects of three major therapeutic groups contributing to cardio-renal-metabolic protection. Abbreviations: SGLT-2i, sodium-glucose co-transporter type 2 inhibitors; GLP-1, glucagon-like peptide-1; RA, receptor agonist, GIP, glucose-dependent insulinotropic polypeptide; CKD, chronic kidney disease; MACEs, major adverse cardiovascular events.

A comparative summary of the mechanisms and clinical effects of these cardiometabolic agents is illustrated in Figure 2.

### 4.4. Emerging and Adjunctive Therapies in Cardiorenal Protection

#### 4.4.1. Finerenone (Selective Non-Steroidal Mineralocorticoid Receptor Antagonist)

Finerenone is the first non-steroidal mineralocorticoid receptor antagonist (MRA) to demonstrate dual renal and cardiovascular protection in patients with T2DM and CKD. In the FIDELIO-DKD and FIGARO-DKD trials, finerenone significantly reduced kidney-disease progression and cardiovascular events when added to optimized renin-angiotensin system blockade, with fewer hyperkalemia-related discontinuations than steroidal MRAs [77,78]. A recent network meta-analysis including 12 studies involving finerenone confirmed its favorable efficacy and safety profile, showing a modest but significant reduction in systolic blood pressure (mean difference = −1.65 mmHg; 95% CI −2.48 to −0.81) compared with placebo. Among the broader comparison with SGLT2 inhibitors and GLP-1 receptor agonists, finerenone ranked among the agents with the lowest incidence of urinary-tract infection, highlighting its tolerability in patients with CKD [79]. Finerenone’s antifibrotic and anti-inflammatory properties, together with its renal and cardiovascular benefits, make it a valuable adjunct to SGLT2 inhibition in patients with T2DM and CKD.

#### 4.4.2. Sacubitril/Valsartan (Angiotensin-Receptor Neprilysin Inhibitor, ARNI)

Sacubitril/valsartan combines neprilysin inhibition with angiotensin-II receptor blockade, enhancing natriuretic-peptide signaling while counteracting RAAS overactivation. The PARADIGM-HF trial established its superiority over enalapril in reducing mortality and heart-failure hospitalizations in patients with HF with reduced ejection fraction [80], whereas PARAGON-HF extended the therapeutic spectrum toward HF with preserved ejection fraction [81]. A recent meta-analysis of 14 randomized trials including ≈ 25,000 patients confirmed these findings: sacubitril/valsartan reduced all-cause mortality in patients with EF ≤ 40% (RR 0.88; 95% CI 0.81–0.94) and lowered HF rehospitalization rates across the EF spectrum (RR 0.85; 95% CI 0.79–0.91), although no significant reduction in CV-specific mortality was observed [82]. These data consolidate ARNI as a foundational therapy for HF with reduced ejection fraction and a preferred option in selected HF with mild or preserved ejection fraction populations.

Together, finerenone and sacubitril/valsartan exemplify the ongoing convergence of cardiovascular, renal, and metabolic therapeutics broadening the treatment landscape beyond glucose control toward integrated organ protection, supported by high-level evidence.

## 5. Novel Anti-Thrombotic Strategies

### 5.1. Ischemic Heart Disease (ACS, CAD, PAD)

Over the past two decades, antithrombotic therapy in ischemic heart disease has undergone a profound transformation. Once a rigid, standardized approach, it has progressively evolved into more flexible and individualized strategies, balancing ischemic protection with bleeding risk. In acute coronary syndromes (ACS), antiplatelet therapy has progressed significantly. Ticagrelor and prasugrel have progressively replaced clopidogrel, providing faster, more potent, and more consistent platelet inhibition, as demonstrated in the PLATO, ISAR-REACT 5, and TRITON-TIMI 38 trials [83,84,85].

Equally transformative has been the advent of de-escalation strategies. The TWILIGHT and TICO trials showed that shortening dual antiplatelet therapy (DAPT) and continuing with P2Y12 inhibitor monotherapy significantly reduces bleeding without compromising ischemic protection [86,87,88,89]. However, therapy duration should be tailored to each patient’s ischemic and bleeding profile rather than fixed.

Beyond coronary disease, the concept of dual-pathway inhibition has expanded the therapeutic horizon. The COMPASS trial demonstrated that very-low-dose rivaroxaban (2.5 mg twice daily) combined with aspirin reduces MACE in patients with coronary artery disease (CAD) or peripheral artery disease (PAD), albeit with an increase in nonfatal bleeding [90]. The VOYAGER-PAD trial confirmed similar benefits in patients undergoing peripheral revascularization [91]. Altogether, these data support a paradigm shift toward shorter, safer, and more personalized antiplatelet strategies across the ischemic spectrum.

### 5.2. Atrial Fibrillation

In atrial fibrillation (AF), the most recent ESC (2024) and ACC/AHA/ACCP/HRS (2023) consensus documents firmly establish direct oral anticoagulants (DOACs) as the standard of care over vitamin K antagonists (VKAs), due to their predictable pharmacokinetics, lack of routine monitoring, and significant reduction in intracranial hemorrhage [92,93,94,95]. Among these, apixaban has emerged as a cornerstone. In the ARISTOTLE trial, apixaban reduced stroke, systemic embolism, major bleeding, and mortality compared with warfarin [96].

Its role was further strengthened by the AUGUSTUS trial, where apixaban combined with a P2Y12 inhibitor (without aspirin) in patients with AF undergoing PCI reduced bleeding while preserving ischemic efficacy [97]. Based on this evidence, guidelines now recommend a very short course of triple therapy (usually one week, extended up to one month in patients at very high ischemic risk), followed by dual therapy and eventually oral anticoagulant monotherapy [92,93,94,95].

Confidence in DOACs has also been reinforced by the availability of specific reversal agents, such as idarucizumab for dabigatran (RE-VERSE AD) and andexanet alfa for factor Xa inhibitors (ANNEXA-4, ANNEXA-I), which allow rapid reversal in cases of major bleeding or urgent surgery [98,99,100].

### 5.3. Venous Thromboembolism (VTE)

The management of VTE has similarly benefited from the evolution of DOAC-based regimens. Large-scale randomized trials such as AMPLIFY, EINSTEIN-DVT/PE, and Hokusai-VTE have demonstrated that DOACs are at least as effective as standard VKA-based therapy while offering a lower risk of major bleeding and greater ease of use [101,102,103,104]. These findings have simplified long-term anticoagulation for both treatment and secondary prevention, supporting broader adoption of DOACs as first-line agents across the VTE spectrum.

### 5.4. Emerging and Future Directions

Several innovative strategies are currently under investigation. Factor XI/XIa inhibitors, designed to dissociate thrombotic protection from bleeding risk, have generated robust interest across multiple programs [100,105]. Early proof-of-concept studies using an antisense oligonucleotide and monoclonal antibodies (e.g., FXI-ASO, osocimab, abelacimab) in orthopedic prophylaxis (FOXTROT) demonstrated effective prevention of venous thromboembolism with minimal bleeding [106,107,108]. More recently, oral small-molecule inhibitors have expanded the field. Asundexian showed favorable safety and marked FXIa suppression in phase II trials of atrial fibrillation (PACIFIC-AF) [109], acute myocardial infarction on DAPT (PACIFIC-AMI) [110], and non-cardioembolic stroke (PACIFIC-STROKE) [111]. However, the phase III OCEANIC-AF trial demonstrated inferiority of asundexian compared with apixaban for stroke prevention in AF [112], while OCEANIC-STROKE remains ongoing (NCT05686070). Milvexian has shown encouraging results in phase II studies for secondary stroke prevention (AXIOMATIC-SSP) [113] and post-arthroplasty thromboprophylaxis [105] and is currently being tested in the large phase III LIBREXIA-ACS trial [114]. Additionally, the monoclonal antibody abelacimab demonstrated substantial reductions in bleeding versus rivaroxaban in AF in the AZALEA-TIMI 71 trial, further supporting the haemostasis-sparing hypothesis of FXI/XIa inhibition [115]. Collectively, these data reinforce the biological rationale for this pathway while underscoring the need for ongoing phase III validation across diverse thromboembolic settings.

Another promising field is pharmacogenomic guidance of antiplatelet therapy. The POPULAR Genetics trial showed that CYP2C19 genotyping can guide the choice between clopidogrel and ticagrelor in STEMI patients undergoing PCI [116,117], although the TAILOR-PCI trial did not confirm a clear clinical benefit [118]. These approaches underscore the growing interest in genetic and molecular tailoring of antithrombotic therapy, though further validation is required before routine implementation [119,120].

### 5.5. Comparative Analysis

The comparative evaluation of antithrombotic agents underscores how therapeutic choices increasingly depend on clinical context and patient profile rather than a universal hierarchy. In ACS, ticagrelor demonstrated superiority over clopidogrel in the PLATO trial [83], while prasugrel proved more effective than ticagrelor in NSTEMI patients undergoing PCI in ISAR-REACT 5, though its use is restricted in elderly, low-weight patients, and those with prior stroke/TIA [84]. Monotherapy strategies with ticagrelor, tested in TWILIGHT and TICO, confirmed that early aspirin withdrawal can lower bleeding without loss of ischemic protection [86,87,88].

Among anticoagulants, apixaban has emerged as the leading DOAC. In ARISTOTLE, it reduced stroke, systemic embolism, major bleeding, and mortality compared with warfarin [96]. In AUGUSTUS, apixaban plus a P2Y12 inhibitor (without aspirin) lowered bleeding in AF patients undergoing PCI, with preserved ischemic efficacy [97]. These results support current guidelines that recommend a very short course of triple therapy (1 week, up to 1 month in high ischemic risk), followed by dual therapy and finally OAC monotherapy [92,93,94]. Novel approaches provide further contrasts. Dual-pathway inhibition with very-low-dose rivaroxaban plus aspirin, validated in COMPASS and VOYAGER-PAD, offers incremental ischemic protection in CAD/PAD, though at the cost of more nonfatal bleeding [90,91]. Conversely, enthusiasm for factor XI/XIa inhibitors has been tempered by the negative OCEANIC-AF trial, which showed inferiority of asundexian versus apixaban in AF, leaving these agents without a defined role [112]. Altogether, current evidence supports a precision-based approach where the optimal antithrombotic regimen balances efficacy against bleeding risk and aligns with each patient’s indication, comorbidities, and risk profile. The trajectory of antithrombotic therapy is thus moving toward shorter, less intensive, and more individualized regimens, bringing clinical practice closer to truly personalized cardiovascular care (Table 1 and Table 2).

## 6. New Therapeutic Strategies in Cardiovascular Disease

The multifactorial pathogenesis and clinical heterogeneity of CVD often limit the effectiveness of traditional pharmacological therapies. In recent years, innovative strategies have emerged, including gene therapy, RNA-based approaches, nanomedicine, and digital health technologies [129]. These modalities aim to correct molecular defects, modulate pathogenic pathways, and pave the way toward precision medicine through patient-specific interventions.

### 6.1. Gene Therapy and CRISPR-Cas9 Strategies

Gene therapy in CVD aims to correct the functional consequences of pathogenic variants. Two main approaches have been described:

- Correction of dominant-negative effects: In conditions where a mutated allele produces toxic proteins that interfere with normal function, the therapeutic goal is selective suppression of the mutant gene. This can be achieved by allele-specific inactivation through CRISPR/Cas9 nucleases, which induce double-strand breaks repaired by NHEJ or, less efficiently, HDR [130,131]. Alternatively, transcript silencing with antisense oligonucleotides (ASOs) or siRNAs allows reversible suppression, though requiring repeat administration [132]. While CRISPR offers permanent correction, RNA-targeted therapies provide dose-dependent, reversible control that may be safer in specific contexts.
- Correction of haploinsufficiency: When disease results from insufficient protein production due to inactivation of one allele, the therapeutic goal is to restore protein levels. This can be achieved by exogenous gene delivery with AAV vectors, already tested in models of cardiomyopathies such as Danon and Fabry disease [133], or by genome editing using base and prime editors [134]. These tools correct point mutations in post-mitotic cardiomyocytes without double-strand breaks, showing promise in murine models of hypertrophic and dilated cardiomyopathy [135].

A major challenge remains in vivo cardiac delivery. AAV vectors are widely used but limited by cargo capacity, immunogenicity, and dose-related toxicity [136]. Strategies under investigation include compact Cas9 orthologs, dual-vector systems, self-complementary AAVs, and engineered capsids [137]. Non-viral alternatives, such as lipid nanoparticles and virus-like particles, are less immunogenic and scalable, but cardiac efficiency remains limited [138,139]. Long-term safety is another critical issue. Off-target editing is addressed with high-fidelity Cas variants, optimized sgRNA design, and unbiased detection methods [140]. Early clinical trials, such as NTLA-2001 for transthyretin amyloidosis, confirm the feasibility of in vivo editing [141], but translation into cardiology will require rigorous evaluation, extended follow-up, and careful ethical oversight.

### 6.2. MicroRNA and Non-Coding RNA-Based Therapies

Epitranscriptomics has revealed the crucial role of RNA modifications (m6A, m5C, m1A) in regulating gene expression and contributing to cardiovascular pathophysiology [142,143]. In parallel, non-coding RNAs (miRNAs, lncRNAs, circRNAs) have emerged as key regulators and therapeutic targets. The miRNAs such as miR-21, implicated in fibrosis and hypertrophy, and miR-29, associated with post-infarction remodeling, represent promising candidates, although inconsistencies remain in the literature [143,144,145,146]. LncRNAs such as CAREL and Wisper regulate cardiomyocyte proliferation and fibroblast activation; antisense oligonucleotides targeting profibrotic lncRNAs have shown efficacy in vivo [147,148,149]. CircRNAs, such as CDR1as, act as miRNA sponges, modulating cardiac injury responses [150]. Challenges include tissue-specific delivery, off-target effects, and immunogenicity. However, advances such as chemical modifications, GalNAc conjugation, and lipid nanoparticles have improved RNA stability and uptake, facilitating the development of cardiac-targeted ASOs. Several RNA-based therapies have already entered early-phase clinical trials [151,152,153].

Zilebesiran is a subcutaneous small-interfering RNA (siRNA) molecule that targets hepatic angiotensinogen mRNA, leading to durable suppression of circulating angiotensinogen and sustained blood pressure reduction with infrequent dosing [154]. In early- and mid-phase randomized trials, Zilebesiran produced clinically meaningful reductions in both office and ambulatory blood pressure, with a favorable safety profile. A recent systematic review and meta-analysis of two randomized controlled trials confirmed these effects, showing a mean 24 h systolic BP reduction of −15.1 mmHg (95% CI −17.2 to −13.0) and diastolic BP reduction of −7.3 mmHg (95% CI −8.7 to −6.0) at 12 weeks compared with placebo, with no between-study heterogeneity (I^2^ = 0%) [155]. The incidence of total adverse events was modestly higher versus placebo (RR 1.15, 95% CI 1.01–1.30), driven mainly by mild injection-site reactions and headache, while serious adverse events did not differ. Renal safety was supported by stable serum creatinine and estimated GFR across studies [155]. However, current evidence remains limited by small sample size and under-representation of non-white populations; larger phase III outcome trials are warranted to determine long-term efficacy and safety.

### 6.3. Nanomedicine and Targeted Drug Delivery

Nanomedicine is emerging as a promising approach in cardiovascular therapy, using nanoscale carriers to improve solubility, pharmacokinetics, and tissue specificity of drugs and genetic materials [156]. Lipid nanoparticles (LNPs), validated through mRNA vaccines, are being adapted to deliver siRNA, mRNA, and CRISPR-based agents for disorders such as hyperlipidemia and transthyretin amyloidosis [155,156,157]. Other platforms include polymeric nanoparticles, based on biodegradable polymers, and inorganic nanostructures such as gold particles, which allow controlled release and enhanced stability [158]. Targeting strategies have advanced with surface ligands that guide nanoparticles to inflamed endothelium or ischemic myocardium, and stimuli-responsive systems that release drugs in acidic or enzymatic environments, thereby maximizing local action while reducing systemic toxicity [159,160]. Preclinical studies support applications across major cardiovascular conditions. In atherosclerosis, nanoparticles deliver anti-inflammatory molecules, antioxidants, and gene-silencing agents to stabilize plaques [161,162]. In myocardial infarction and HF, they transport cardioprotective and regenerative factors to damaged myocardium [163,164]. In amyloidosis, LNP-mediated siRNA or CRISPR delivery to the liver reduces pathogenic protein production [165]. While promising, clinical translation is still limited by concerns about long-term biocompatibility, large-scale reproducibility, and regulatory complexity. Nevertheless, the integration of nanomedicine with RNA- and gene-based technologies is accelerating progress toward precision cardiovascular therapeutics.

### 6.4. Digital Health: Artificial Intelligence and Big Data

Digital health technologies, including mobile apps, wearable sensors, and telemedicine platforms, integrated with Big Data and artificial intelligence (AI), are reshaping cardiovascular care.

- Diagnosis and risk stratification: AI applied to ECG and imaging improves arrhythmia detection and tissue characterization [166,167].
- Personalized medicine: AI-driven clinical decision support systems integrated into EHRs provide real-time recommendations [16].
- Remote monitoring: wearable devices enable continuous follow-up, facilitating early detection of HF or arrhythmias and reducing hospitalizations [168].
- Drug discovery: AI accelerates the identification of therapeutic targets and drug candidates [169].

The translation of these technologies into clinical practice still requires rigorous validation, interoperability, and careful regulation. Yet, the convergence of gene therapy, RNA-based therapeutics, nanomedicine, and digital health is reshaping cardiovascular medicine, moving from symptomatic treatment toward truly mechanism-based, personalized care. Precision cardiology is no longer a distant goal, but an emerging reality.

## 7. Adherence, Personalization, and Economic Considerations

### 7.1. Challenges in Treatment Adherence

Adherence to long-term cardiovascular therapies is essential for treatment success, yet it remains a persistent challenge worldwide. According to the WHO, between 40% and 50% of patients with chronic diseases fail to follow prescribed regimens as recommended [170,171]. The reasons are multiple: forgetfulness, fear of side effects, low motivation due to perceived lack of efficacy, limited health literacy, and skepticism toward medical advice. Structural barriers such as high out-of-pocket costs or inadequate communication with healthcare providers further complicate the picture [172]. The consequences are considerable. Poor adherence accounts for about 10% of all hospital admissions and generates $100–300 billion in preventable healthcare expenditures annually in the United States alone [173]. These costs reflect disease progression that could have been avoided, unnecessary diagnostic procedures, and emergency visits that strain healthcare systems. Improving adherence requires more than simply educating patients, it calls for a patient-centered approach that builds trust and reduces complexity. Vulnerable groups such as the elderly, patients with multimorbidity, or those with limited resources need particular support. Strategies that simplify therapy, promote shared decision-making, and integrate digital tools (apps, reminders, remote monitoring) can make adherence more achievable [174]. Closing the adherence gap is therefore not only a clinical target but also a step toward more equitable cardiovascular care.

### 7.2. Innovative Formulations and Strategies

A major driver of adherence is treatment simplicity. Traditional regimens with multiple daily doses are difficult to sustain, especially in chronic care. Innovative formulations and delivery systems are helping to bridge this gap.

Long-acting therapies. Inclisiran, a small interfering RNA against PCSK9, requires only two subcutaneous injections per year, offering ~50% LDL-C reduction in ORION trials [175,176]. Long-acting GLP-1 receptor agonists and once-daily SGLT2 inhibitors also improve convenience while delivering consistent cardiometabolic protection [177,178].

Fixed-dose combinations. The “polypill” strategy, combining statins, antihypertensives, and antiplatelets into a single pill, has proven effective in reducing pill burden and improving outcomes. In the SECURE trial, post-MI patients treated with a cardiovascular polypill experienced fewer recurrent events compared to standard therapy [179].

Frontier approaches. Gene-editing strategies such as CRISPR-Cas9 targeting PCSK9 or ANGPTL3 may eventually provide one-time, durable LDL-C lowering, while nanoparticle-based delivery systems are being developed to optimize tissue targeting and prolong drug release [180,181].

Digital solutions. Mobile apps, smart pill dispensers, and wearable biosensors can support adherence in daily life, offering real-time reminders and generating useful data for clinicians [129].

These tools are particularly valuable for patients with multimorbidity or cognitive impairment. Overall, innovations that simplify treatment schedules, reduce pill burden, and integrate digital support are not only clinically effective but also more compatible with the reality of long-term patient care.

### 7.3. Economic and Regulatory Perspectives

The promise of innovation in cardiovascular therapeutics is closely tied to its affordability and accessibility. Many of the most advanced treatments (e.g., monoclonal antibodies, RNA-based drugs, and gene therapies) carry high costs that limit their use [182]. For example, the cost per quality-adjusted life year gained with PCSK9 inhibitors has been estimated between $150,000 and $450,000, depending on patient risk and national pricing policies [183]. By contrast, SGLT2 inhibitors, though more expensive than generics, are considered cost-effective in high-risk patients due to reductions in HF hospitalizations and renal events [184]. Global inequalities in access remain a concern. While high-income countries can provide early access to innovative therapies, low- and middle-income countries face limited availability due to pricing, reimbursement, and healthcare infrastructure. Such disparities risk widening the gap in cardiovascular outcomes across populations. Regulatory agencies are adapting, with fast-track and breakthrough designations accelerating the approval of innovative drugs, often based on surrogate endpoints [185]. This makes post-marketing surveillance and real-world evidence essential to confirm long-term efficacy and safety. Health Technology Assessment bodies and payers are increasingly incorporating real world evidence into reimbursement decisions. New economic models, such as value-based pricing and risk-sharing agreements, are emerging to manage uncertainty and improve affordability [186]. By linking reimbursement to real-world outcomes, these approaches can help align innovation with sustainability. Ultimately, the challenge is to ensure that scientific progress translates into clinical benefit for all patients. Balancing efficacy, adherence, cost-effectiveness, and equitable access will be key to shaping a cardiovascular care system that is both innovative and sustainable.

## 8. Conclusions and Future Perspectives

Cardiovascular medicine is undergoing a transformative shift driven by the convergence of pharmacological, genetic, and digital innovations. The integration of next-generation lipid-lowering agents, metabolic drugs with pleiotropic effects, and refined antithrombotic strategies has already redefined prevention and treatment paradigms. Meanwhile, RNA-based therapeutics, CRISPR-mediated editing, and nanomedicine are moving from proof-of-concept research to early clinical translation, offering the prospect of durable, mechanism-based interventions. In parallel, artificial intelligence and digital health platforms are reshaping clinical decision-making, risk stratification, and patient monitoring, creating a truly data-driven ecosystem of care.

Future progress will depend on addressing several key challenges. First, the generation of long-term outcome and safety data for novel agents, especially gene and RNA therapies, is essential to ensure their integration into clinical practice. Second, real-world evidence should complement trial data to evaluate the effectiveness, adherence, and cost–benefit balance of innovative treatments in heterogeneous populations. Third, the interoperability of digital systems and the ethical governance of patient data must be strengthened to fully exploit artificial intelligence without compromising privacy or equity. Finally, equitable access remains a moral and policy priority: innovation must not widen the gap between high- and low-resource settings.

The next decade is likely to see cardiovascular therapeutics evolve from disease-specific management toward a unified, precision-based model combining molecular correction, system-level analytics, and personalized adherence support. The ultimate goal will be not only to prolong survival, but to deliver sustainable cardiovascular health through the seamless integration of pharmacological, genetic, and digital frontiers.

## Figures and Tables

**Table 1:** Comparative overview of established and emerging antithrombotic therapies in cardiovascular disease.

Drug/Class	Mechanism of Action	Main Clinical Trials	Key Findings	Administration	Safety Profile	Current/Investigational Status
Ticagrelor	Reversible P2Y_12_ receptor antagonist	PLATO, TWILIGHT, TICO	↓ MACE vs. clopidogrel; effective as monotherapy after short DAPT	Oral, BID	↑ Bleeding vs. clopidogrel, ↑ dyspnea	Approved for ACS and post-PCI
Prasugrel	Irreversible P2Y_12_ receptor antagonist	TRITON-TIMI 38, ISAR-REACT 5	Superior to clopidogrel, especially in PCI; avoid in ≥75 yrs or low body weight	Oral, daily	Major bleeding risk	Approved for ACS undergoing PCI
Clopidogrel	Irreversible P2Y_12_ receptor antagonist	CURE, CAPRIE	Standard agent; reduced effect in CYP2C19 loss-of-function	Oral, daily	Lower bleeding risk	Generic standard
Apixaban	Factor Xa inhibitor	ARISTOTLE, AUGUSTUS, AMPLIFY	↓ Stroke, major bleeding, mortality vs. warfarin; effective for VTE treatment/prevention	Oral, BID	Lower ICH risk	Approved for AF, VTE, post-PCI dual therapy
Rivaroxaban	Factor Xa inhibitor	COMPASS, VOYAGER-PAD, EINSTEIN-DVT/PE	Dual-pathway inhibition ↓ MACE/PAD events with aspirin; effective for VTE	Oral, daily/BID	↑Non-fatal bleeding	Approved for AF, CAD/PAD, VTE
Dabigatran	Direct thrombin inhibitor	RE-LY, RE-VERSE AD	↓ Stroke vs. warfarin; reversal with idarucizumab	Oral, BID	Dyspepsia, GI bleeding	Approved for AF/VTE
Factor XI/XIa inhibitors	Block intrinsic coagulation pathway upstream of thrombin	FOXTROT, PACIFIC-AF/AMI/STROKE, OCEANIC-AF/STROKE, AXIOMATIC-SSP, LIBREXIA-ACS, AZALEA-TIMI 71	Promising phase II; asundexian inferior to apixaban in phase III	Oral (investigational)	Lower bleeding in early trials	Early studies show effective VTE prevention; asundexian safe in phase II but inferior to apixaban in OCEANIC-AF; abelacimab ↓ bleeding vs. rivaroxaban; milvexian promising in stroke and post-ACS settings
Dual-pathway inhibition	Very-low-dose rivaroxaban + aspirin	COMPASS, VOYAGER-PAD	↓ MACE, limb ischemia; ↑ non-fatal bleeding	Oral combination	Manageable bleeding	Recommended for high-risk CAD/PAD

Abbreviations: ACS, acute coronary syndrome; DAPT, dual antiplatelet therapy; PCI, percutaneous coronary intervention; MACE, major adverse cardiovascular events; AF, atrial fibrillation; VTE, venous thromboembolism; CAD, coronary artery disease; PAD, peripheral artery disease; ICH, intracranial hemorrhage; GI, gastrointestinal; BID, twice daily; CYP2C19, cytochrome P450 2C19; ↑ increased; ↓ decreased.

**Table 2:** Current guideline recommendations for major cardiometabolic and cardiovascular therapies.

Drug/Class	Principal Indications	Guideline Recommendation (Class/Level of Evidence)	Primary Source
PCSK9 inhibitors (alirocumab, evolocumab)	Persistent LDL-C elevation despite max statin ± ezetimibe	Class I/Level A-reduce ASCVD events	[121]
Inclisiran	Alternative/add-on LDL-C lowering therapy for very-high-risk pts not at target	Class IIa/Level A	[121]
Bempedoic acid	Statin-intolerant or insufficient response on statin ± ezetimibe	Class IIa/Level A	[121]
SGLT2 inhibitors	HF (HFrEF, HFmrEF, HFpEF); T2DM + CKD	Class I/Level A-reduce HF hospitalization, CV & renal events	[122,123,124]
GLP-1 receptor agonists	T2DM + ASCVD/high CV risk	Class I/Level A-MACE reduction	[125]
Tirzepatide	T2DM and obesity	Approved; outcome data emerging	[125]
Finerenone	T2DM + CKD with albuminuria on ACEi/ARB	Class I/Level A-reduce renal & CV events	[123]
Sacubitril/Valsartan (ARNI)	HFrEF	Class I/Level B (ESC); I/A (ACC/AHA)-reduce mortality & HF hospitalization	[122,124]
	HFmrEF/HFpEF (selected)	Class IIa/Level B-symptom & HF hospitalization benefit	[122]
DOACs (e.g., Apixaban, Rivaroxaban)	Non-valvular AF; VTE	Class I/Level A-preferred over VKA	[92,94]
Dual-pathway inhibition (rivaroxaban 2.5 mg bid + aspirin)	Symptomatic PAD (high ischemic risk)	Class IIa/Level A-B-reduce MACE & MALE with acceptable bleeding risk	[126,127]
Antiplatelet therapy (Aspirin + P2Y_12_ inhibitor)	ACS and post-PCI secondary prevention	Class I/Level A-DAPT (aspirin + ticagrelor/prasugrel preferred over clopidogrel); duration 12 months, shorter (3–6 months) if high bleeding risk	[93,128]
P2Y_12_ monotherapy after short DAPT	Post-PCI, high bleeding risk	Class IIa/Level A-early aspirin withdrawal (ticagrelor or prasugrel monotherapy)	[93]

Abbreviations: ACEi, angiotensin-converting enzyme inhibitor; ARB, angiotensin receptor blocker; ARNI, angiotensin receptor neprilysin inhibitor; ASCVD, atherosclerotic cardiovascular disease; CKD, chronic kidney disease; CV, cardiovascular; DOAC, direct oral anticoagulant; ESC, European Society of Cardiology; HF, heart failure; HFrEF, heart failure with reduced ejection fraction; HFmrEF, heart failure with mildly reduced ejection fraction; HFpEF, heart failure with preserved ejection fraction; MACE, major adverse cardiovascular events; MALE, major adverse limb events; PAD, peripheral artery disease; SGLT2, sodium-glucose cotransporter-2; T2DM, type 2 diabetes mellitus; VKA, vitamin K antagonist.

## Data Availability

No new data were created or analyzed in this study. Data sharing is not applicable to this article.
